# The augmented expression of the cytidine deaminase gene by 5-azacytidine predicts therapeutic efficacy in myelodysplastic syndromes

**DOI:** 10.18632/oncotarget.26784

**Published:** 2019-03-19

**Authors:** Yuichi Murakami, Yoshizo Kimura, Akihiko Kawahara, Shohei Mitsuyasu, Hidetoshi Miyake, Kaoru Tohyama, Yoshio Endo, Nao Yoshida, Yutaka Imamura, Kosuke Watari, Mayumi Ono, Takashi Okamura, Michihiko Kuwano

**Affiliations:** ^1^ Cancer Translational Research Center, St. Mary's Institute of Health Sciences, Kurume, Japan; ^2^ Department of Pharmaceutical Oncology, Graduate School of Pharmaceutical Sciences, Kyushu University, Fukuoka, Japan; ^3^ Department of Pathology, St. Mary’s Hospital, Kurume, Japan; ^4^ Department of Diagnostic Pathology, Kurume University Hospital, Kurume, Japan; ^5^ Department of Pharmacy, St. Mary's Hospital, Kurume, Japan; ^6^ Department of Laboratory Medicine, Kawasaki Medical School, Okayama, Japan; ^7^ Central Research Resource Branch, Cancer Research Institute, Kanazawa University, Kanazawa, Japan; ^8^ Department of Hematology, St. Mary's Hospital, Kurume, Japan; ^9^ Hematology and Oncology Center, St. Mary’s Hospital, Kurume, Japan

**Keywords:** myelodysplastic syndromes, cytidine deaminase, 5-azacytidine, DNA methylation, hypomethylating agent

## Abstract

5-Azacytidine (5AC), a hypomethylating agent, is clinically used for the treatment of patients with myelodysplastic syndromes (MDS). Cytidine deaminase (CDA) is a key enzyme in the detoxification of 5AC. We investigated whether the CDA expression could predict response to 5AC in MDS. Among leukemia-derived cell lines, MDS-L, an MDS-derived cell line with a relatively low CDA expression level, was found to be the most sensitive to 5AC. Combination with tetrahydrouridine, an inhibitor of CDA, synergistically potentiated the cytotoxic effect of 5AC. Treatment with 5AC markedly enhanced the expression level of CDA mRNA and showed demethylation at CpG sites in the 5′-flanking region of the CDA gene. We further compared the protein expression levels of CDA in matched clinical samples before and after treatment with 5AC in bone marrow cells from 8 MDS patients by an immunohistochemical analysis. The CDA expression level showed an approximately 2- to 3-fold increase after 5AC treatment in 3 of these cases, and these three patients with relatively higher CDA expression levels after 5AC treatment all showed better clinical responses to 5AC. In contrast, the 5 remaining patients, whose CDA expression showed no augmentation, observed no clinical benefit. Taken together, the optimized determination of the CDA expression levels before and after 5AC treatment, and the methylation status at CpG sites of 5′-flanking region of the CDA gene, may contribute to the development of precise 5AC therapy for MDS.

## INTRODUCTION

Myelodysplastic syndromes (MDS) is a group of heterogenous hematopoietic stem cell disorders, resulting in cytopenia and an increased risk of progression into acute myeloid leukemia. MDS patients have a high frequency of cytogenetic abnormalities. The gene mutation of epigenetic regulators, including ten-eleven translocation 2, isocitrate dehydrogenase 1/2, DNA methyltransferase 3A, additional sex combs like 1 and enhancer of zeste homolog 2, have been implicated in the pathogenesis of MDS [1–3]. The DNA methylation profiles of MDS patients are distinct from healthy individuals [4]. Epigenetic deregulation, such as gene hypermethylation and histone deacetylation, plays a key role in the progression of MDS [5, 6]. Thus, treatment with hypomethylating agents has been expected to improve the efficacy of treatments targeting MDS.

**Figure 1:**
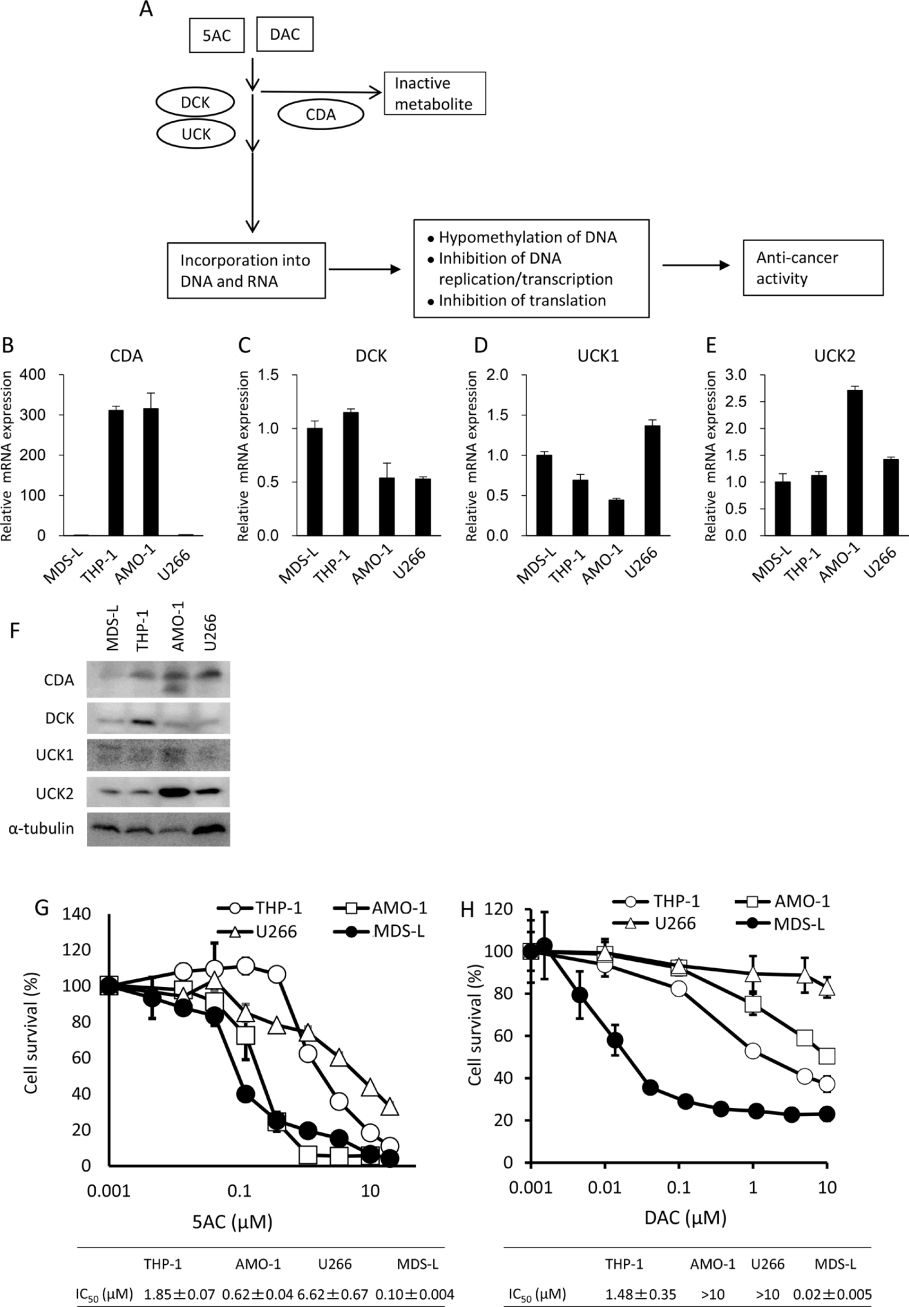
An MDS-derived cell line, MDS-L, is highly susceptible to cytotoxicity from DNA hypomethylating agents (**A**) A schematic representation of the metabolic pathways of DNA hypomethylating agents, 5AC and DAC. (**B**–**E**) The mRNA expression levels of CDA, DCK, UCK1 and UCK2 in hematopoietic tumor cell lines were determined by a quantitative RT-PCR. Relative mRNA levels were normalized by ribosomal RNA. The expression level in MDS-L was set to 1. (**F**) Western blot analysis of metabolic molecules of 5AC in MDS-L, THP-1, AMO-1 and U266. (**G** and **H**) The dose response curves of THP-1, AMO-1, U266 and MDS-L to various doses of 5AC (G) and DAC (H) when exposed for 3 days. Each value represents the mean ± SD of triplicate wells. IC50 values are presented for each cell line.

In clinical trials of 5-azacytidine (5AC), the most important cytostatic action was first highlighted against MDS [7]. The cytidine analogs 5AC and 5-aza-2′-deoxycytidine (DAC), which are hypomethylating agents, were approved for use in the treatment of MDS, and are effective in terms of overall survival [8–10]. 5AC and DAC are translocated into cells by diffusion or membrane transporter mediated influx, are phosphorylated to triphosphate (active form) by uridinecytidine kinase (UCK) and deoxycytidine kinase (DCK), and are finally incorporated into the DNA or RNA, leading to aberrant DNA replication, genome instability, aberrant transcription and translation, and epigenetic changes (Figure 1A). On the other hand, 5AC and DAC are pharmacologically inactivated by cytidine deaminase (CDA), a key enzyme catalyzing cytidine deamination (Figure 1A). The relative ratio of the CDA expression to the DCK expression has been shown to be higher in MDS patients who are non-responders to DAC [11]. A relevant study demonstrated that when treated with 5AC or DAC, the CDA enzyme activity of male MDS patients was higher than that of female MDS patients; furthermore, these male patients showed worse overall survival [12].

In this study, we first investigated whether the expression levels of CDA were associated with the responsiveness of MDS cells to 5AC. Secondly, we investigated whether the expression of CDA is upregulated in response to 5AC and whether 5AC modifies the methylation status in the 5′-flanking region of the CDA gene. Taken together, the altered CDA expression level after 5AC treatment predicts the therapeutic responsiveness of MDS patients.

## RESULTS

### MDS-derived cells show relatively higher sensitivity to the cytotoxic effects of 5AC or DAC

Figure 1A shows a pharmacologically metabolic model of how CDA detoxifies 5AC and DAC, and how DCK or UCK stimulate the incorporation of 5AC and DAC into DNA and/or RNA, resulting in the exertion of their cytotoxicity effects on cancer cells.

We first examined the CDA, DCK, UCK1 and UCK2 expression levels in hematopoietic tumor cells (MDS-L, THP-1, AMO-1 and U266 cells). Of the four cell lines, the expression levels of CDA mRNA were higher in THP-1 and AMO-1 when there was low expression in MDS-L and U266 (Figure 1B). All four cell lines showed comparable levels of 2- to 3-fold differences in both DCK and UCK1 in mRNA (Figure 1C and 1D). By contrast, AMO-1 showed relatively higher levels of UCK2 mRNA than other three lines (Figure 1E). Western blot analysis showed the lowest expression of CDA protein in MDS-L (Figure 1F), consistent with its mRNA levels (Figure 1B). THP-1, AMO-1 and U266 showed similar expression levels of CDA protein, but U266 showed much less mRNA levels of CDA than other two lines (Figure 1B and 1F). The expression level of DCK protein was relatively higher in THP-1 than other three cell lines and all four cell lines showed similar levels of UCK1 protein (Figure 1F). Of the four cell lines, AMO-1 showed the highest expression levels of UCK2 (Figure 1F), consistent with its mRNA level (Figure 1E).

The dose response curves to 5AC and DAC showed that MDS-L was the most sensitive to the cytotoxicity of 5AC and DAC among these cell lines (Figure 1G and 1H). Further AMO-1 also showed higher sensitivity to 5AC than other two lines, but not to DAC (Figure 1G and 1H).

### CDA annihilates the cytotoxicity of 5AC in MDS cells

**Figure 2:**
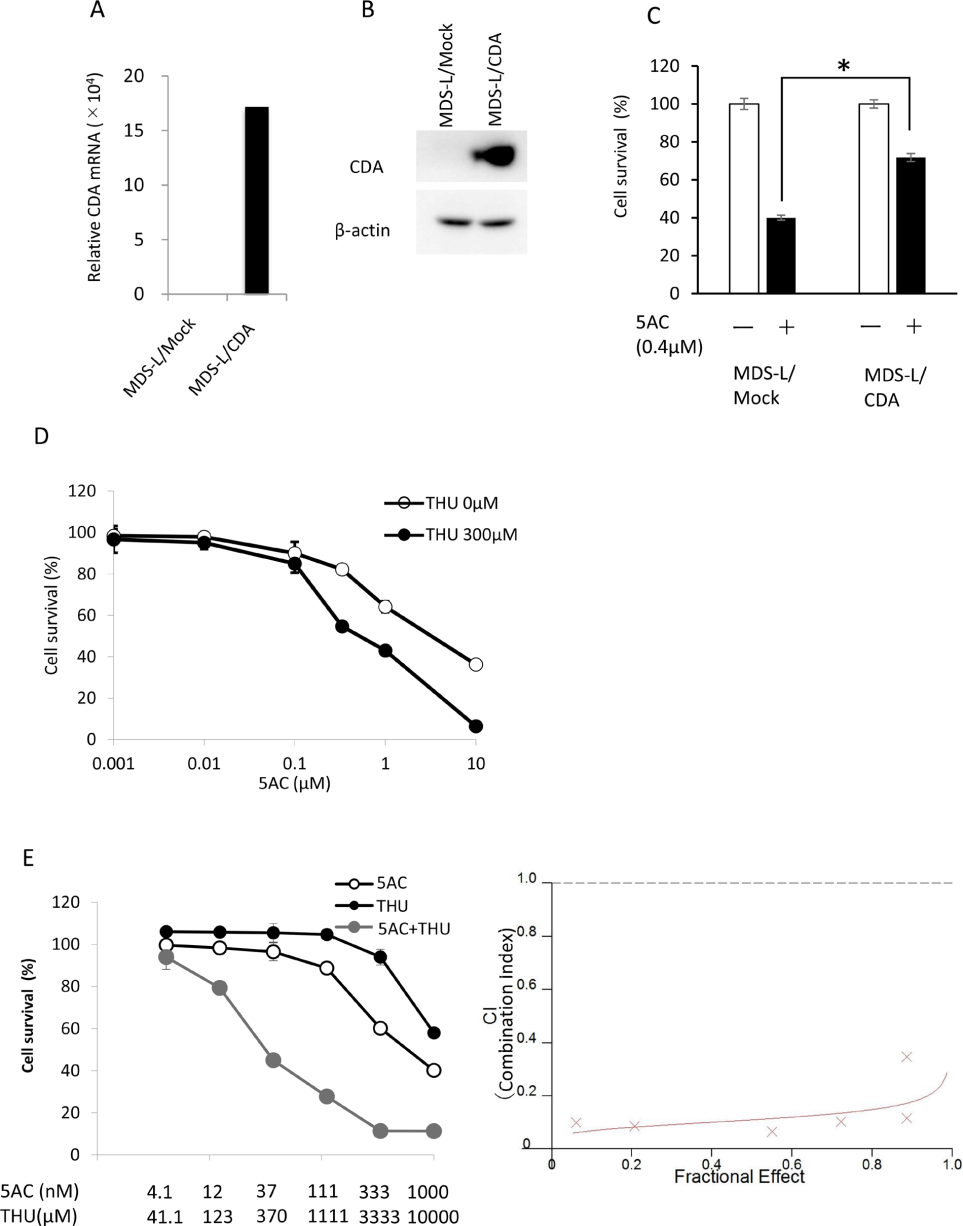
The expression of CDA was closely associated with cellular sensitivity to 5AC (**A**) The CDA mRNA expression levels in MDS-L/Mock and MDS-L/CDA cells were determined by a quantitative RT-PCR. (**B**) The CDA protein expression levels in MDS-L/Mock and MDS-L/CDA cells were determined by Western blotting. (**C**) The sensitivity of MDS-L/Mock and MDS-L/CDA cells to 5AC. Cells were exposed to 0.4µM 5AC for 72 h and subjected to a WST assay. Each value represents the mean ± SD of triplicate wells. ^*^p < 0.01 (**D**) The sensitivity of MDS-L/CDA cells to 5AC in combination with 300 µM THU. The cells were exposed to 5AC and THU for 72 h and subjected to a WST assay. Each value represents the mean ± SD of triplicate wells. (**E**) The combination index (CI) of 5AC and THU in MDS-L/CDA cells. The dose-response curves of single 5AC, single THU and their combination were determined by a WST assay (left). The cells were exposed to 5AC and THU for 72 h. The CI values were calculated according to Chou-Talay’s method (right).

Since CDA is a key enzyme that protects against the cytotoxicity of 5AC (Figure 1A), we established MDS-L cells overexpressing CDA (MDS-L/CDA) using a lentivirus vector, and MDS-L/CDA cells showed higher expression levels of both the mRNA and protein of CDA in comparison to MDS-L/Mock cells (Figure 2A and 2B). MDS-L/CDA cells showed relatively less sensitivity to the cytotoxic effects of 5AC than MDS-L/Mock cells (Figure 2C). We next examined whether combination with a CDA inhibitor, tetrahydrouridine (THU), could further potentiate the cytotoxic effect of 5AC in MDS-L/CDA cells (Figure 2D). A combination index (CI) analysis by the Chou-Tarlalay method [13] was performed to characterize the combination effect of 5AC and THU. In this analysis, CI values greater than 1 were considered antagonistic, CI values equal to 1 were considered additive, and CI values less than 1 were considered synergistic. All of the CI values were less than 1 when 5AC was combined with THU (Figure 2E), indicating that the combination of 5AC and THU induces synergistic cytotoxicity in MDS cells.

### 5AC induces the expression of the CDA gene and the demethylation of CpG sites at the 5′-flanking region of the CDA gene

**Figure 3:**
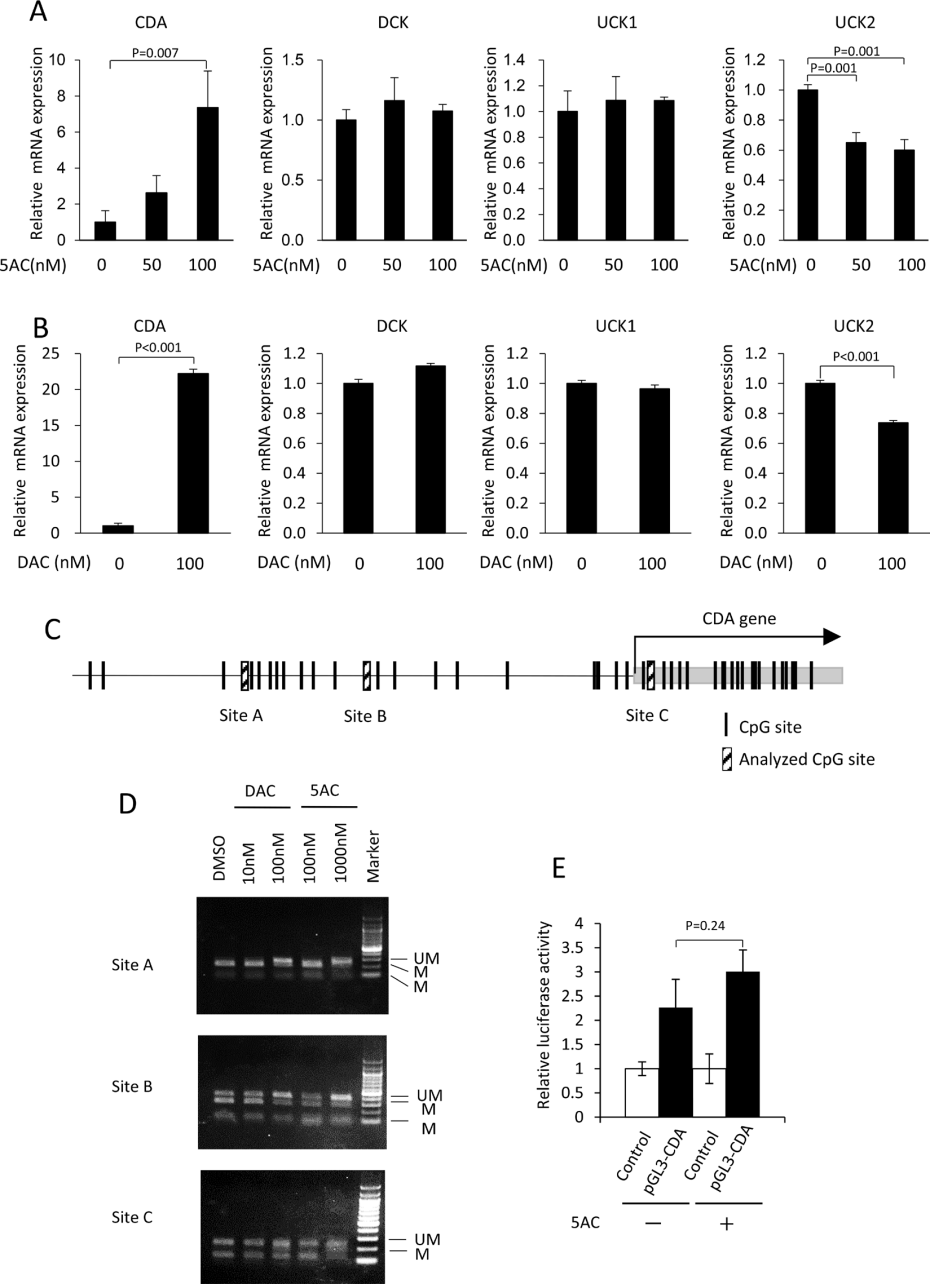
Treatment with 5AC induced the expression of CDA, together with the demethylation of the CpG site in the 5′-flanking region of the CDA gene (**A**) The mRNA expression levels of CDA, DCK, UCK1 and UCK2 in MDS-L cells after treatment with various doses of 5AC for 48 h were determined by a quantitative RT-PCR. Each mRNA level was normalized to the rRNA level, and the value relative to that in untreated cells was shown. The expression level in untreated cells was set to 1. (**B**) The mRNA expression levels of CDA, DCK, UCK1 and UCK2 in MDS-L cells after treatment with DAC for 48 h were determined. Each mRNA level was normalized to the rRNA level, and the value relative to that in untreated cells was shown. The expression level in untreated cells was set to 1. (**C**) A schematic representation of the potential CpG sites in the 5′-flanking region of the CDA gene. (**D**) The effect of DAC or 5AC on the methylation status of the 5′-flanking region of CDA gene. Cells were treated with DAC or 5AC for 48 h. The (UM) and (M) bands represent the unmethylated and methylated bands, respectively. (**E**) Comparison of luciferase activity driven by CDA promoter. The cells were treated with 5AC at 100 nM for 48 h. The relative promoter activity was presented as ratio to the activity in control vector-transfected cells.

We examined whether the CDA mRNA levels were augmented by 5AC or DAC. 5AC induced an approximately 7-fold enhancement in the CDA mRNA levels (Figure 3A). In contrast, there were no apparent changes in the mRNA levels of DCK and UCK1 after treatment with 5AC. The UCK2 mRNA expression was slightly, but significantly, decreased after treatment with 5AC. Treatment with DAC also induced marked increases in CDA mRNA levels, but not mRNA levels of DCK and UCK1 genes (Figure 3B). The expression of UCK2 mRNA was also slightly, but significantly decreased after DAC treatment. Among the genes relevant to the metabolism of DNA demethylating agents, the expression of CDA is markedly augmented when treated with 5AC and DAC.

We next searched the CpG sites in 5′-flanking region of the CDA gene, and found that three CpG sites (A, B and C) in the CDA 5′-flanking region were methylated in MDS-L cells (Figure 3C). Of the three CpG methylation sites, sites A and B were both demethylated, but there was no apparent change at site C, after treatment with DAC at 100 nM or 5AC at 1000 nM (Figure 3D). DAC or 5AC therefore induces the enhanced expression of the CDA gene, and the demethylation of the CpG site in the 5′-flanking region of the CDA gene in MDS cells.

To determine whether 5AC induces transcriptional activation of CDA under non-methylated condition, we further examined luciferase reporter assay using CDA promoter vector. 5AC treatment did not enhance the luciferase activity in CDA promoter vector-transfected MDS-L cells (Figure 3E). Together, 5AC as well as DAC induces CDA expression through the epigenetic change of the CpG site in the 5′-flanking region of the CDA gene.

### The immunohistochemical analysis of CDA and DCK in tumor samples before and after treatment with 5AC

**Figure 4:**
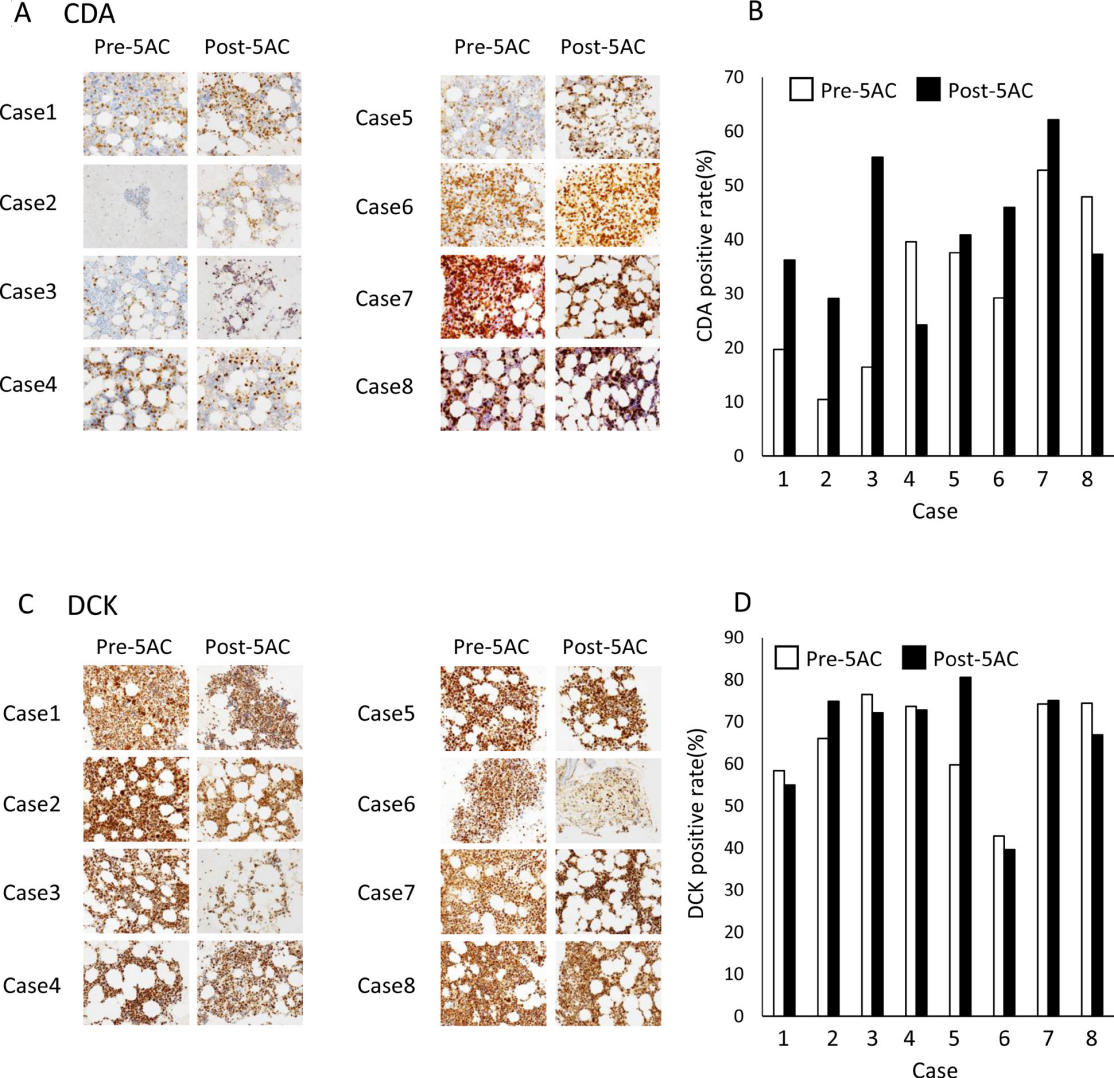
The IHC analysis of clinical samples from patients with MDS (**A**) The IHC analysis of CDA in bone marrow biopsy samples from patients with MDS before (Pre-5AC) and after (Post-5AC) treatment. (**B**) The quantitative analysis of the CDA expression levels in patients with MDS before and after 5AC treatment, based on the IHC analysis. (**C**) The IHC analysis of DCK in bone marrow biopsy samples from patients with MDS before and after 5AC treatment. (**D**) The quantitative analysis of the DCK expression in patients with MDS before and after 5AC treatment, based on the IHC analysis.

We finally examined the CDA and DCK expression levels by IHC in bone marrow biopsy samples from MDS patients who had been treated with 5AC. The clinical outcomes of eight patients are presented in Table 1 and cases 1, 2 and 3 showed a clinical response. Figure 4A shows representative IHC images of 8 clinical samples before (pre-5AC) and after (post-5AC) treatments. It is noteworthy that an approximately 1.5- to 4-fold increase in the expression of CDA was observed in 4 (case 1, 2, 3 and 6) of 8 cases after treatment with 5AC (Figure 4B). However, there was no apparent increase in the CDA expression levels after 5AC treatment in the other 4 clinical samples. On the other hand, the DCK expression before and after 5AC treatment was similar in all eight clinical samples (Figure 4C and 4D).

**Table 1:** The characteristics and therapeutic status of MDS patients during 5AC treatment

Case	Age/Sex	Diagnosis	IPSS	Tissue	Therapy	Responses		Outcomes
						Early response^a)^	Late response^b)^	
No.1	80/M	RAEB-2^c)^	High	BM	5AC x7	CR	CR	Died/Pneumonia
No.2	64/M	RAEB-1^c)^	Int2	BM	5AC x6 -> SCT^g)^	PR	PD	Died/regimen-related toxicity
No.3	60/M	RA^d)^∼MDS-U	Int2	BM	5AC x5 ->SCT^g)^	PR	NA^h)^	Alive
No.4	76/M	RAEB-1^c)^	Int1	BM	5AC x6	SD	SD	Alive
No.5	61/M	RCMD^e)^	Int2	BM	5AC x6	PD	PD	Died/Pneumonia
No.6	78/M	CMML^f)^	Int1	BM	5AC x6	PD	PD	Died/Cause-specific death
No.7	59/F	RAEB-2^c)^	Int2	BM	5AC x8	PD	PD	Died/Cause-specific death
No.8	65/M	RAEB-2^c)^	High	BM	5AC x8	SD	SD	Died/Pneumonia

a)Responses after treatment with 5AC 3 times

b)Responses after treatment with 5AC 6 times

c)RAEB: refractory anemia with excess blasts

d)RA: refractory anemia

e)RCMD: refractory cytopenia with multilineage dysplasia

f)CMML: chronic myelomonocytic leukemia

g)Stem cell transplantation (SCT) following the initial 5AC therapy.

h)Not available

### The methylation status in the 5′-flanking region of the CDA gene in MDS

**Figure 5:**
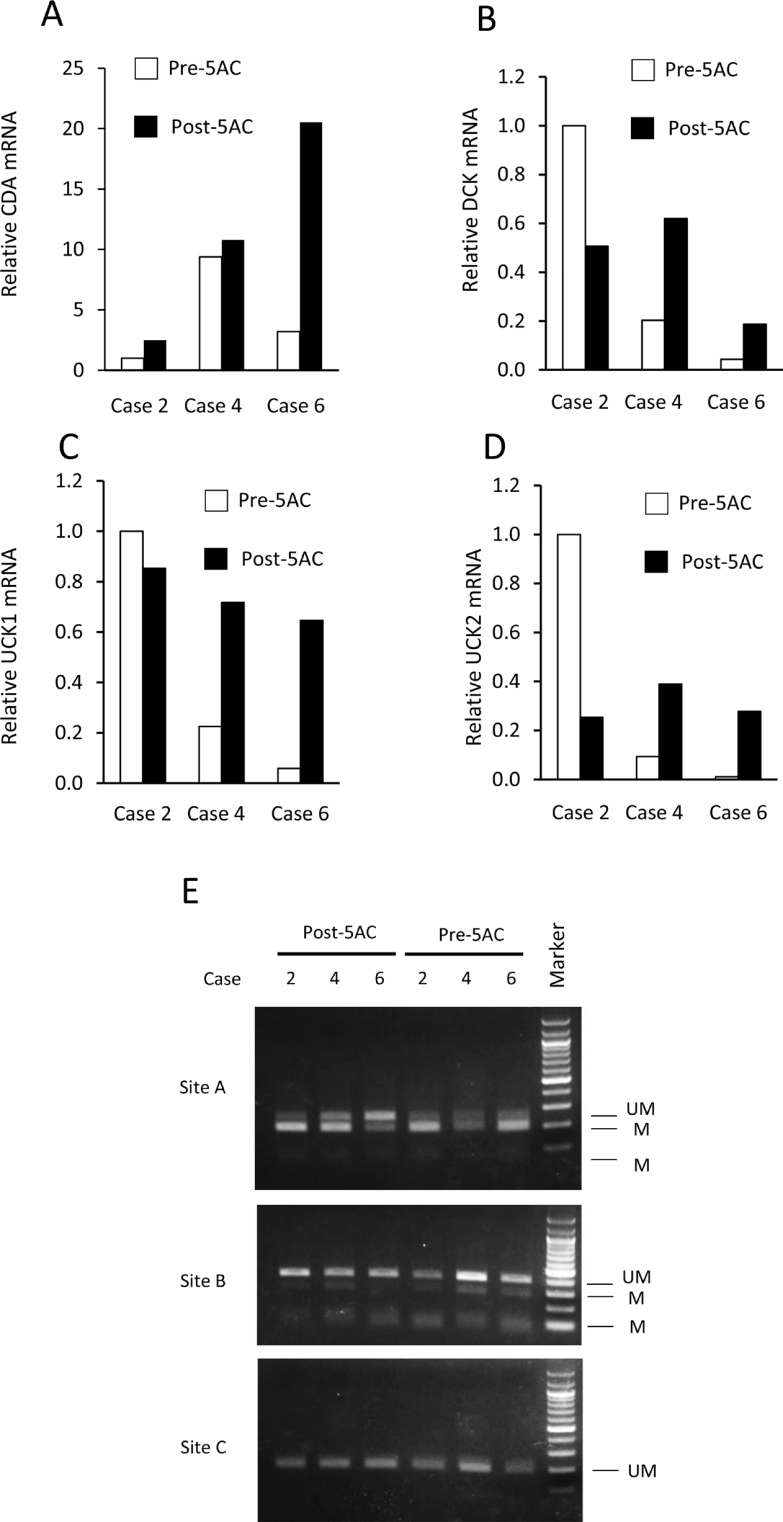
The expression levels of CDA, DCK, UCK1 and UCK2 mRNA and the methylation status at the 5′-flanking regions of the CDA gene before and after 5AC treatment in three matched clinical samples (**A**–**D**) The mRNA expression levels of CDA (A), DCK (B), UCK1 (C) and UCK2 (D) in tumor samples of patients with MDS before (Pre-5AC) and after (Post-5AC) treatment were determined by a quantitative RT-PCR. The expression level in Pre-5AC samples of case 2 was set to 1 (**E**) The methylation status of the 5′-flanking region of the CDA gene in clinical samples from three patients with MDS before (Pre-5AC) and after (Post-5AC) treatment (cases 2, 4 and 6). The (UM) and (M) bands represent the unmethylated and methylated bands, respectively. The CpG sites that were analyzed are shown in Figure 3C.

We further examined the expression of CDA mRNA in bone marrow biopsy samples from MDS patients using three matched and frozen clinical samples (cases 2, 4 and 6). The CDA, DCK, UCK1 and UCK2 mRNA expression levels are shown in clinical samples obtained before and after 5AC treatment in three cases (Figure 5A–5D). In agreement with the results of the IHC analysis (Figure 4A), the CDA expression levels in cases 2 and 6 were higher after 5AC treatment, while the pre- and post-treatment levels of case 4 were similar (Figure 5A). The DCK, UCK1 and UCK2 mRNA expression levels were increased after 5AC treatment in cases 4 and case 6 and decreased in case 2 (Figure 5B–5D).

The methylation status of the CpG sites (sites A, B and C) (see Figure 3C) was further determined by an analysis of frozen samples of three matched cases in cases 2, 4 and 6 (Figure 5E). In case 6, hypomethylation was observed at site A after 5AC treatment, while there was no apparent change in the methylation status at site A in cases 2 and 4 (Figure 5E). The methylation status at sites B and C was unchanged in all three cases.

## DISCUSSION

CDA is a representative detoxification enzyme of anticancer cytidine analogs such as gemcitabine, cytarabine, DAC and 5AC, and CDA activity limits the therapeutic efficacy of such cytidine analogs (see Figure 1A). The overexpression of CDA has been previously reported to induce resistance to cytarabine and gemcitabine in hematopoietic progenitor cells [14]. Pharmacokinetic and pharmacodynamic studies on the deaminase inhibitor THU consistently presented the idea that THU increases DNMT1 inhibition by DAC [15]. Combination with THU markedly enhanced the chemosensitivity of pancreatic cancer cells to gemcitabine [16].

In our present study, MDS-L, an MDS-derived cell line [17], was highly sensitive to the cytotoxicity of 5AC and DAC, when MDS-L showed low expression of CDA mRNA and protein. Both THP-1 and U266, which showed relatively higher expression of CDA mRNA and protein of the tumor cell lines used, are resistant to 5AC and DAC. In contrast, AMO-1, which showed high expression level of CDA protein, was sensitive to the cytotoxicity to 5AC, but not DAC. Since AMO-1 showed the highest expression of UCK2 mRNA and protein of the four cell lines, the sensitivity to 5AC in AMO-1 might be correlated with high expression of UCK2 involved the incorporation of 5AC into DNA and/or RNA (Figure 1). However, this is still highly speculative until further elucidation on the relevant mechanism.

The overexpression of CDA in MDS cells conferred decreased sensitivity to 5AC (Figure 2C), and THU synergistically potentiated 5AC in MDS cells (Figure 2D and 2E). CDA polymorphism (79A>C) is significantly correlated with shorter overall survival in comparison to wild-type CDA in AML patients [18], and a high CDA/DCK ratio is a risk factor for resistance to DAC in MDS patients [11]. Collectively, CDA plays key role, not only in the toxicity of anticancer cytidine analogs (including 5AC and DAC), but also in the limitation of the anticancer therapeutic efficacy of these agents in patients with MDS or other malignancies.

Aberrant DNA methylation is often induced in the early stage of MDS, suggesting that aberrant DNA methylation is a mechanism for tumor suppressor gene silencing as MDS evolves into AML [19]. Surprisingly, 5AC specifically induced the marked upregulation of the CDA gene; this was accompanied by hypomethylation at the 5′-regulatory sites associated with the expression of the CDA gene in MDS cells (Figure 3). The expression of the CDA gene is therefore highly susceptible to 5AC, indicating the close coupling of the cytidine analog with the expression of the CDA gene.

Uchida et al. reported that 5AC was administered at 75 mg⁄m^2^ once daily for 7 days on a 28-day cycle to MDS patients and the plasma C_max_ was 1120 ng/mL (4.59 µM) [20]. In our present study, 5AC at 50 to 100 nM induced expression of CDA in MDS-L cells (Figure 3A), and the plasma C_max_ of 5AC is thus estimated to be enough to upregulates expression of CDA in patients. We showed that the expression levels of CDA in bone marrow biopsy samples were increased after 5AC treatment in four cases (cases 1, 2, 3 and 6) when eight matched cases were analyzed by IHC analysis (Figure 4A and 4B). Of the four cases, three cases (cases 1, 2, and 3) showed a clinical response (CR and PR) (Table 1). Collectively, based on our IHC analysis, a relative increase in the CDA expression level after 5AC treatment may predict responsiveness to 5AC in MDS.

With regard to the possible correlation of the enhanced expression of CDA mRNA with demethylation at the CpG methylation sites of its promoter region, we examined the mRNA levels and methylation status of CDA using lyophilized tumor samples of three matched cases (cases 2, 4 and 6). Cases 2 and 6, which showed early responsiveness (CR and PD, respectively) after 5AC treatment, showed an approximately 3-fold and 6-fold increase in their CDA mRNA expression levels, respectively (Figure 5), which was consistent with the IHC data (Figure 4). CpG site A was hypomethylated after 5AC treatment in case 6 (Figure 5E). Whether the determination of the CDA mRNA levels and/or methylation status could be useful for predicting the clinical responses of MDS patients should be further investigated using frozen and matched tumor samples obtained at optimized periods during 5AC treatment.

CDA inactivates 5AC, a potent anticancer agent that is widely used in the treatment of MDS, and treatment with 5AC induces the enhanced expression of the CDA gene in MDS cells, which is accompanied by the altered methylation of the CpG sites at its 5′-flanking region. IHC showed the enhanced expression of CDA in tumor samples after 5AC treatment in patients who were responsive to 5AC treatment. The determination of CDA expression levels may predict the clinical response of MDS patients. However, it should be noted that the present study only represents the preliminary findings in a small number population. The optimized determination of the CDA protein and mRNA expression levels, as well as the methylation status of the CDA gene can be expected to contribute to improving the precision of 5AC therapy in the treatment of patients with MDS.

## MATERIALS AND METHODS

### Cell culture and reagents

AMO-1, U266 and THP-1 cells were cultured in RPMI 1640 supplemented with 10% fetal bovine serum in an atmosphere containing 5% CO2, as described previously [21]. MDS-L cells were cultured in RPMI 1640 supplemented with 10% fetal bovine serum, 50 μM 2-mercaptoethanol and 20 ng/ml IL-3 [17]. These cells were not further tested or authenticated by the authors. 5AC and DAC were purchased from Sigma-Aldrich.

### The construction of the expression vector and lentiviral transduction

Full-length cDNA of human CDA cDNA [22] was subcloned into pCDH-EF1-MCS-BGH-PGK-GFP-T2A-Puro Vector (System Bioscience) to generate a pCDH-CDA construct. Lentiviral particles were produced by co-transfection with pCDH-CDA and pPACKH1 packaging plasmid (System Bioscience) into 293TN cells using Lipofectamine LTX, in accordance with the manufacturer’s instructions. MDS-L overexpressing CDA (MDS-L/CDA) cells were then established by lentiviral infection.

### Quantitative real-time PCR

Total RNA was isolated from cell cultures using ISOGEN reagent (Nippon Gene Co. Ltd., Tokyo, Japan) according to the manufacturer’s instructions, as described previously [23]. The primer pairs and probes (CDA, Hs00156401_m1; DCK, Hs01040726_m1; UCK1, Hs1075618_m1; UCK2, Hs0089900_m1) were obtained from Applied Biosystems. The thermal cycle conditions included maintaining the reactions at 48° C for 15 min and at 95° C for 10 min, and then alternating for 40 cycles between 95° C for 15 s and 60° C for 1 min. The relative gene expression for each sample was determined using the formula 2̂(-delta Ct) = 2̂(Ct(18S ribosomal RNA)– Ct(target), which reflected the target gene expression normalized to the18S ribosomal RNA level.

### Western blotting

Western blotting was performed as described previously [23]. Cells were rinsed with ice-cold PBS and lysed in buffer containing 50 mmol/L HEPES, 120 mmol/L NaCl, 50 mmol/L NaF, 1% Triton X-100, 10% glycerol, 5 mmol/L EDTA, 1 mmol/L phenylmethylsulfonyl fluoride, 10 μg/mL aprotinin, 10 μg/mL leupeptin, and 1 mmol/L sodium orthovanadate. Cell lysates were separated by SDS-PAGE and transferred to Immobilon membranes (Millipore Corp.). After transfer, the membranes were incubated in blocking solution and probed with anti-CDA (Abcam), DCK (Abcam), UCK1 (Santacruz Biotechnology, Inc.), α-tubulin (Sigma) or anti-β-actin (Abcam) antibodies. The intensity of luminescence was quantified using an image-analysis system (LAS-4000 mini; GE healthcare).

### Combined bisulfite restriction analysis

Bisulfite modification of genomic DNA was performed using a Methyl Easy Xceed Rapid DNA Bisulphite Modification Kit (TAKARA BIO, Inc.) according to the manufacturer’s instructions, as described previously [23]. Briefly, DNA was denatured by NaOH (final concentration 0.3 µM) for 15 min at 37° C. The samples were then treated with sodium bisulfite solution and were incubated at 80° C for 45 min. Bisulfite-modified DNA was purified according to the protocol, and samples were heat shocked at 95° C for 20 min. A PCR was carried out using the obtained DNA. The sequences of the primers were as follows: site A forward: 5′- TAATTTTAGGTTATTTGGGAGGTTG-3′, site A reverse: 5′-AACCCTTAACACCTTCAAAACTCTT-3′, site B forward: 5′-TATATTTGGTTTGAAAAAGTTTTAGAATTA-3′, site B reverse: 5′-AAAAAAAACAAAACAATATCTC-3′, site C forward: 5′-TTTTTAAGGGAGAGTGTGAAGTATA-3′, site C reverse: 5′-AACCTAAACTAAAACCCCAAACAC-3′. The PCR products were digested by AciI, then analyzed on 3% agarose gels and stained with ethidium bromide.

### Luciferase assay

For generating pGL3-CDA-luc, the promoter region of CDA gene from position -2338 to +41 was amplified by PCR with the primer pair 5′-ACGTACGCGTTTCTCCTTCCAACCGAATG-3′ and 5′-GTACCTCGAGTTCAGGATGCAGGGTCTAGG-3′. The PCR products were ligated into the MluI-XhoI sites of the pGL3-Basic vector (Promega). pGL3-Basic vector or pGL3-CDA-luc vector was cotransfected along with a renilla luciferase expression vector by electroporation using Amaxa Cell Line Nucleofector Kit L (Lonza). At 6 h after transfection, cells were incubated for further 48 h with 5AC. The luciferase activity was measured by using the dual luciferase assay system according to the manufacturer’s instructions (Promega).

### Patients and tissue samples

A total of eight MDS patients and MDS/myeloproliferative neoplasm (MDS/MPN), who were diagnosed between 2012 and 2016 at the Department of Pathology of St. Mary’s Hospital (Kurume, Japan) were collected in the present study (Table 1). This study was approved by the institutional review board (15-0606), and all patients provided their informed consent in accordance with the Declaration of Helsinki. The clinical and laboratory information of the patients was obtained from the medical records. All patients were treated with 5AC for 3 to 8 cycles and two patients (Cases 2 and 3) received stem cell transplantation following the initial 5AC therapy. Initial 5AC dose was 75 mg/m^2^ body surface area daily for 7 days every 28 days. The dose was adjusted according to hematological toxicities and renal function. The IWG response criteria was used to classify the treatment responses into complete remission (CR), partial remission (PR), stable disease (SD), and progressive disease (PD) [24]. The International Prognostic Scoring System (IPSS) was used to assess the risk of MDS at the initiation of 5AC therapy [25]. Bone marrow tissues were obtained before treatment and on the day after at least 3 cycle 5AC treatment. Paraffin-embedded tissue sections were stained with CDA (ab78231, Abcam, Cambridge, MA, USA) and DCK (LS-B1825, Life Span Bio Sciences, Inc, Seattle, WA, USA) polyclonal antibodies. Antibody dilution and antigen retrieval procedures were performed as standard in the departments concerned.

### Data analysis and filter criteria

The specimens were scanned using an Aperio ScanScopeCS2 (Leica Biosystems/Leica Micrisystems KK) and were analyzed using the Aperio ImageScope software program (Aperio Technologies, Inc.). Basically, the percentages of all nucleated bone marrow cells (ANCs) that were positively stained for each antibody were counted at ×20 magnification to determine the immunohistological score of 0–100%.

c)RAEB: refractory anemia with excess blasts

## Abbreviations

MDS: Myelodysplastic syndromes
5AC: 5-azacytidine
DAC: 5-aza-2′-deoxycytidine
UCK: uridinecytidine kinase
DCK: deoxycytidine kinase
CDA: cytidine deaminase
MPN: myeloproliferative neoplasm
CR: complete remission
PR: partial remission
SD: stable disease
PD: progressive disease
IPSS: The International Prognostic Scoring System
ANC: all nucleated bone marrow cell
THU: tetrahydrouridine
CI: combination index
